# Physiotherapy Practice Patterns for Management of Patients Undergoing Thoracic Surgeries in India: A Survey

**DOI:** 10.1155/2016/9717489

**Published:** 2016-10-31

**Authors:** Sagarika Sultanpuram, Gopala Krishna Alaparthi, Shyam Krishnan Krishnakumar, Zulfeequer C. P. Ottayil

**Affiliations:** Department of Physiotherapy, Kasturba Medical College, Manipal University, Bejai, Mangalore 575004, India

## Abstract

*Aim*. The aim of the current study is to determine the practice patterns of physiotherapists for patients undergoing thoracic surgeries in India.* Materials and Methodology*. A cross-sectional survey was conducted across India in which 600 questionnaires were sent in emails to physiotherapists. The questionnaire addressed assessment and treatment techniques of thoracic surgery.* Results*. A total of 234 completed questionnaires were returned with a response rate of 39%, with the majority of responses received from Telangana, Karnataka, and Andhra Pradesh. More than 90% of the responders practiced physical examination, chest expansion, chest X-ray, ABG analysis, pulmonary function test, and SpO_2_ (oxygen saturation) as the assessment measures in both the pre- and the postoperative phase. Breathing exercises, incentive spirometry, thoracic expansion exercises, coughing and huffing, positioning, and modified postural drainage are found to be commonly used physiotherapy interventions, both pre- and postoperatively, with a response rate of more than 90%. A response rate of more than 84.6% indicated that patients are made to dangle their lower limbs over the edge of the bed on the 1st postoperative day. Mobilization, such as walking up to a chair, sit to stand exercises, and perambulation within the patient's room, was started on the 2nd postoperative day, as stated by more than 65% of the physiotherapists. Staircase climbing was started on the 5th postoperative day. The most commonly used functional evaluation prior to discharge was 6-minute walk test. This was, in fact, practiced by 77.4% of the physiotherapists in their clinical settings.* Conclusion*. The most predominantly employed assessment measures included were physical examination, chest expansion, ABG analysis, pulmonary function test, chest X-ray, SpO_2_ (oxygen saturation), peripheral muscle strength, and cardiopulmonary exercise. The physiotherapy interventions most commonly used were breathing exercises, thoracic expansion exercises, incentive spirometry, and coughing and huffing techniques, in both the pre- and the postoperative phase.

## 1. Introduction

Thoracotomy is the most frequently used open procedure in thoracic surgery and is performed primarily for lung resections [1]. Following thoracic surgery, there is overwhelming evidence of changes in lung function and associated clinical manifestations.

The changes in lung function occurring may be both procedure- and patient-related and occur intra- and postoperatively. Many of these changes are expected following surgery and are transient and self-limiting. These changes include characteristics reduction in lung volume which is primarily restrictive in nature, reduction in functional residual capacity, slowing of mucociliary clearance, and abnormalities in gaseous exchange [2].

This leads to postoperative pulmonary complications [PPC] like atelectasis, pulmonary collapse, consolidation, pleural effusion, persistent air leaks, and pneumonia. Other frequently observed postoperative complications are postthoracotomy pain syndrome and ipsilateral reduction in upper extremity range of motion [ROM] and strength [3–5].

Physiotherapy is widely considered to be important in limiting the development of postoperative pulmonary complications, which are associated with significant clinical and economic impact, and in the prevention and treatment of shoulder dysfunction, which has been reported extensively following thoracotomy [6].

The physiotherapy management of patients after major surgery forms the basis of much debate among physiotherapists worldwide [7]. A growing number of studies have investigated both the current practice and the effectiveness of physiotherapy treatments in patients undergoing cardiac and upper abdominal surgery in India.

There is scarcity of retrievable literature regarding pre- and postoperative assessment and treatment for patients undergoing thoracic surgeries in India. The aim of the study was to analyse current practice patterns among physiotherapists in pre- and postoperative assessment and treatment for patients undergoing thoracic surgeries in India.

## 2. Study Procedure

A written approval was obtained from the Institutional Ethics Committee and Scientific Committee of KMC, Mangalore. Upon approval by the committee, a list of hospitals where thoracic surgeries are done was obtained from the NABH (National Accreditation Board of Hospitals and Health Providers) and MCI (Medical Council of India) websites, and a survey was conducted across India in which around 600 questionnaires were sent via email to physiotherapists who were involved in the management of patients undergoing thoracic surgery.

This email included a hyperlink which directed the participants to a webpage with the Informed Consent Form and those consenting to participate could access the questionnaire.

A period of 2 weeks was given to the physiotherapists to fill in the questionnaire in an attempt to ensure good response rate. If no response was obtained within the stipulated period, telephonic or email reminders were sent and the response was awaited for another 2 weeks beyond which nonresponders were excluded from the study.

## 3. Data Analysis

Responses were numerically coded to allow for descriptive summaries and frequency analysis of the data using SPSS version 16. Frequency variables regarding assessment and treatment for thoracic surgery patients were merged in order to create three responses: “always or frequently,” “sometimes,” and “rarely or never.”

## 4. Result

**Figure 1:**
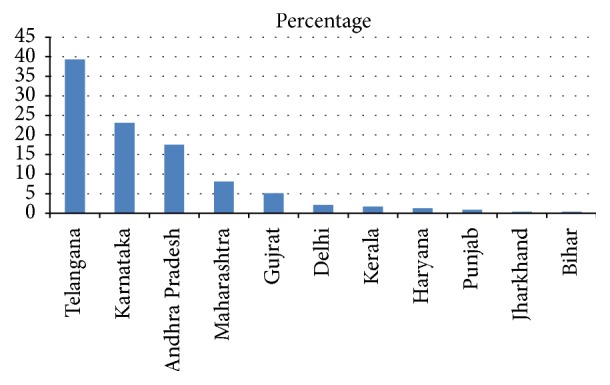
Response rates from different states of India (*n* = 234).

A total of 600 questionnaires were mailed to physiotherapists all over India, of which 234 completed questionnaires were returned. Data are reported as the number of respondents for each individual question, which varied throughout the survey. The physiotherapy profile is displayed in Table 1. The responses were received from 11 states, that is, Telangana, Andhra Pradesh, Karnataka, Bihar, Delhi, Gujarat, Haryana, Jharkhand, Kerala, Maharashtra, and Punjab. This made for a response rate of 42%. The majority of responders were from Telangana (92, 39.35%), Karnataka (54, 23.1%), and Andhra Pradesh (41, 17.5%) and the least responders were from Jharkhand (1, 0.4%) and Bihar (1, 0.4%). Response rate is given in Figure 1.

## 5. Preoperative Assessment for Thoracic Surgery Patients

**Figure 2:**
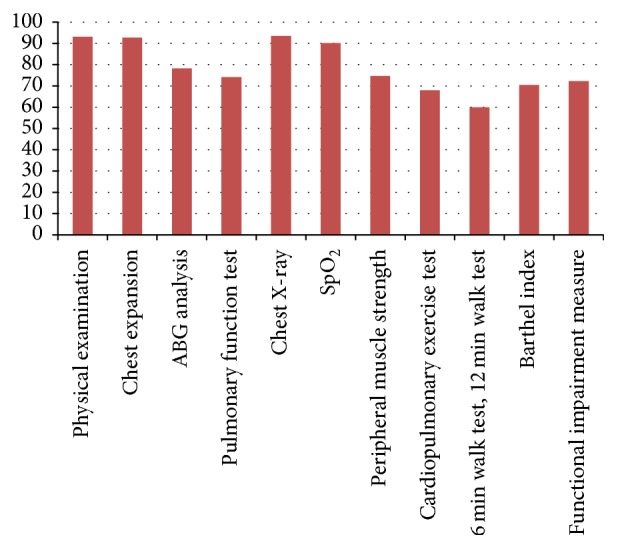
Preoperative assessment for thoracic surgery patients.

The frequencies with which different assessment measures were used preoperatively are given in Table 2 and Figure 2. A total of 99.14% of physiotherapists performed preoperative physiotherapy. Among them, 90% marked “always or frequently.” The various assessment measures included physical examination (*n* = 218  [93.1%]), chest expansion (*n* = 217  [92.7%]), ABG analysis (*n* = 183  [78.2%]), pulmonary function test (*n* = 175  [74.7%]), chest X-ray (*n* = 215  [93.5%]), oxygen saturation (*n* = 211  [90.1%]), peripheral muscle strength (*n* = 175  [74.7%]), cardiopulmonary exercise test (*n* = 159  [67.9%]), 6 min and 12 min walk test (*n* = 138  [60.0%]), Barthel index (*n* = 157  [70.4%]), and functional independence measure (*n* = 164  [72.3%]) of the responders.

## 6. Preoperative Physiotherapy Interventions for Thoracic Surgery Patients

**Figure 3:**
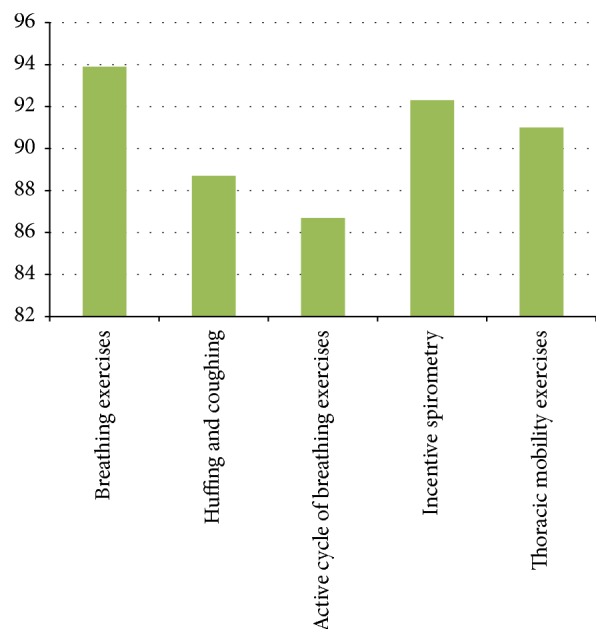
Preoperative physiotherapy interventions for thoracic surgery patients.

The preoperative physiotherapy interventions included deep breathing exercises (*n* = 217  [93.9%]), huffing and coughing (*n* = 204  [88.7%]), active cycle of breathing exercises (*n* = 203  [86.7%]), incentive spirometry (*n* = 216  [92.3%]), and thoracic mobility exercises (*n* = 213  [91.0%]) of the responders (Table 3 and Figure 3).

## 7. Postoperative Assessment for Thoracic Surgery Patients

**Figure 4:**
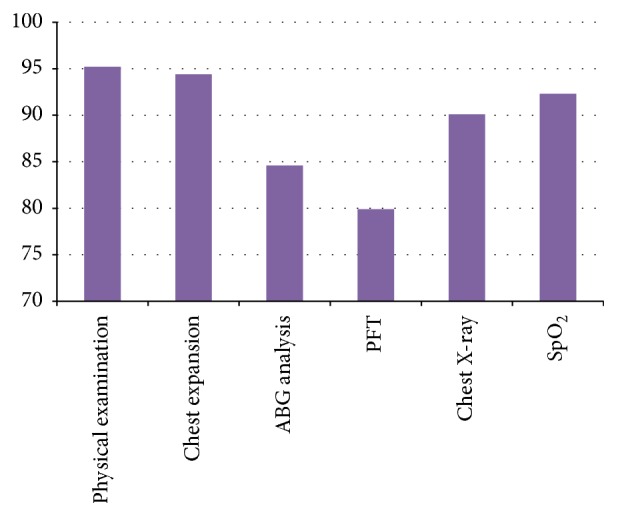
Postoperative assessment for thoracic surgery patients.

The frequencies with which different assessment measures were used postoperatively are given in Table 4 and Figure 4. A total of 90.14% of physiotherapists performed preoperative physiotherapy. Of them, 90% marked “always or frequently.” The various assessment measures included physical examination (*n* = 223  [95.2%]), chest expansion (*n* = 221  [94.4%]), ABG analysis (*n* = 198  [84.6%]), pulmonary function test (*n* = 187  [79.9%]), chest X-ray (*n* = 211  [90.1%]), and oxygen saturation (*n* = 216  [88.4%]).

## 8. Postoperative Physiotherapy Interventions for Thoracic Surgery Patients

### 8.1. Physiotherapy Interventions Used on the 1st Postoperative Day

**Figure 5:**
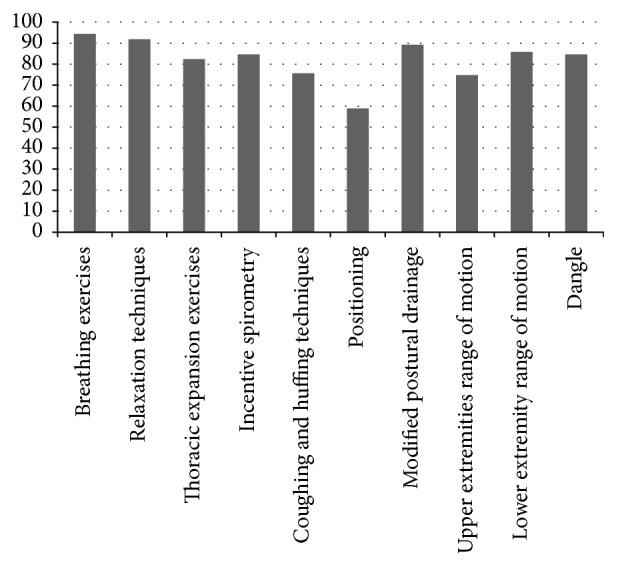
Physiotherapy interventions used on the 1st postoperative day.

The frequencies of various physiotherapy treatment techniques used on postoperative day 1 are given in Table 5 and Figure 5. A total of 97.5% of the physiotherapists performed day 1 rehabilitation. Among them, more than 80% marked “always or frequently.” The treatment techniques included breathing exercises (*n* = 221  [94.4%]), relaxation techniques (*n* = 215  [91.8%]), thoracic expansion exercises (*n* = 193  [82.4%]), incentive spirometry (*n* = 198  [84.6%]), coughing and huffing techniques (*n* = 177  [75.6%]), positioning (*n* = 138  [58.9%]), modified postural drainage (*n* = 206  [88%]), upper extremities range of motion exercises (*n* = 172  [73.5%]), lower extremities range of motion exercises (*n* = 201  [85.8%]), and dangle (*n* = 198  [84.6%]).

### 8.2. Physiotherapy Interventions Used on the 2nd Postoperative Day

**Figure 6:**
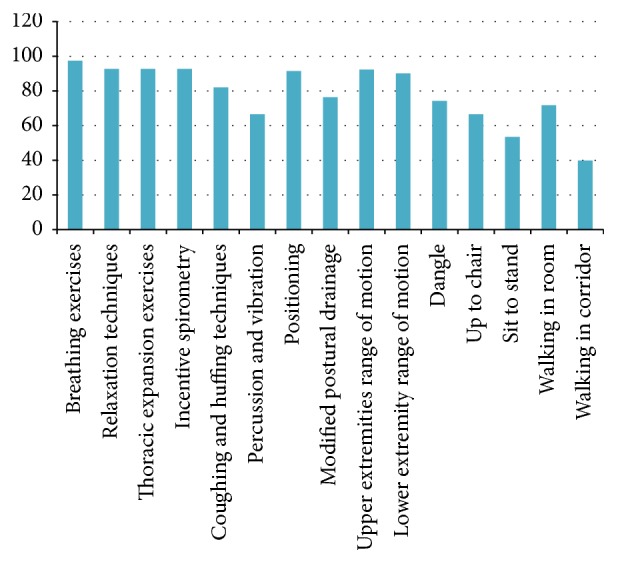
Physiotherapy interventions used on the 2nd postoperative day.

The frequencies with which different treatment techniques were used on postoperative day 2 are given in Table 6 and Figure 6. A total of 97% of the physiotherapists performed day 2 rehabilitation. More than 80% of the physiotherapists marked “always or frequently.” These methods included breathing exercises (*n* = 228  [97.4%]), relaxation techniques (*n* = 217  [92.7%]), thoracic expansion exercises (*n* = 217  [92.7%]), incentive spirometry (*n* = 217  [92.7%]), coughing and huffing techniques (*n* = 192  [82%]), percussion and vibrations (*n* = 156  [66.6%]), positioning (*n* = 214  [91.4%]), modified postural drainage (*n* = 179  [76.4%]), upper extremities range of motion exercises (*n* = 216  [92.3%]), lower extremities range of motion exercises (*n* = 211  [90.1%]), dangle (*n* = 174  [74.3%]), up to chair (*n* = 156  [66.6%]), sit to stand (*n* = 125  [53.4%]), walking in the room (*n* = 168  [71.7%]), and walking in the corridor (*n* = 86  [39.8%]).

### 8.3. Physiotherapy Interventions Used on the 3rd Postoperative Day

**Figure 7:**
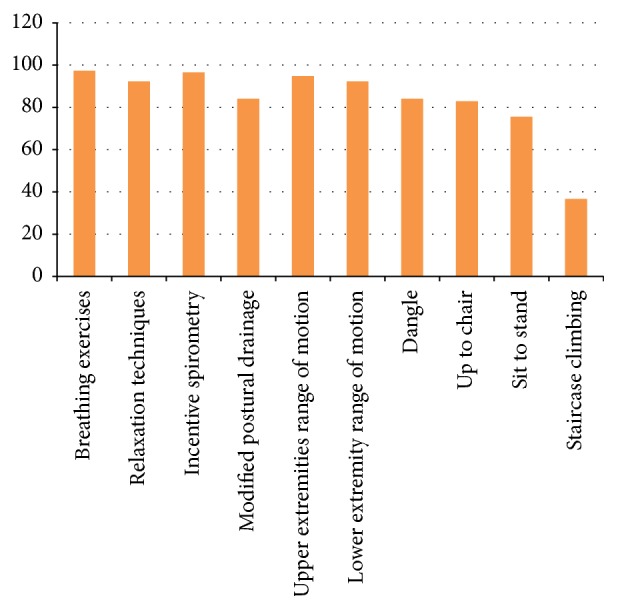
Physiotherapy interventions used on the 3rd postoperative day.

The frequencies with which different treatment techniques were used on postoperative day 3 are given in Table 7 and Figure 7. A total of 98.6% of the physiotherapists performed day 3 rehabilitation. More than 80% of the physiotherapists marked “always or frequently.” The treatment techniques included were breathing exercises (*n* = 228  [97.4%]), relaxation techniques (*n* = 216  [92.3%]), incentive spirometry (*n* = 226  [96.5%]), modified postural drainage (*n* = 197  [84.1%]), upper extremities range of motion exercises (*n* = 222  [94.8%]), lower extremities range of motion exercises (*n* = 216  [92.3%]), dangle (*n* = 197  [84.1%]), up to chair (*n* = 194  [82.9%]), sit to stand (*n* = 177  [75.6%]), and staircase climbing (*n* = 86 [36.7%]) of the responders.

### 8.4. Mobilization on Various Postoperative Days

Data pertaining to mobilization of patients on postoperative days is given in Table 8. A total of 94.4% (*n* = 221) of the physiotherapists responded with data on postoperative day 2. The patients were made to walk 25 to 50 feet by 66.2% (*n* = 155), 60 to 70 feet by 24.7% (*n* = 58), and 80 to 90 feet by 3.5% (*n* = 8); and the patients were made to walk twice daily by 61.1% (*n* = 143), once a day by 1.3% (*n* = 3), thrice daily by 7.3% (*n* = 17), and four times a day by 0.9% (*n* = 2). A total of 99.1% (*n* = 232) of the physiotherapists responded with data on postoperative day 3. The patients were made to walk 110 to 130 feet by 67.5% (*n* = 158), 140 to 160 feet by 24.8% (*n* = 58), 170 to 190 feet by 5.1% (*n* = 12), and >200 feet by 1.7% (*n* = 4) of the responding physiotherapists.

Staircase climbing was started on the 5th postoperative day by 48.3% (*n* = 113); on the 4th postoperative day by 29.1% (*n* = 68); on the 6th postoperative day by 9.8% (*n* = 23); and on the 7th postoperative day by 5.6% (*n* = 13) of the responders. The “functional evaluation prior to discharge” included a 6-minute walk test employed by 77.4% (*n* = 181); a 2-minute walk test employed by 16.7% (*n* = 39); a 12-minute walk test employed by 2.6% (*n* = 6); and a shuttle walk test employed by 0.9% (*n* = 2) of the responders. Discharge teaching was provided by the responders in one of four ways: by an individual [50.9% (*n* = 119)], in written form [27.4% (*n* = 64)], using an audiovisual method [13.2% (*n* = 31)], and by a group [8.1% (*n* = 19)].

## 9. Discussion

To the best of our knowledge, this is the first study reporting on practice patterns adopted by Indian physiotherapists for patients of thoracic surgery. According to the results of our survey, Indian physiotherapists were involved in the assessment and management of patients both pre- and postoperatively.

The novel findings of the study were as follows:

1. The assessment primarily focused on physical examination, chest expansion, ABG analysis, pulmonary function test, chest X-ray, SpO_2_ (oxygen saturation), and peripheral muscle strength. Other impairment measures like cardiopulmonary exercise test, 2 min walk test, 6 min walk test, and 12 min walk test were used by fewer responders in their clinical practice. There was limited use by the responders of functional measures like the Barthel index and functional independence measure.
2. The most commonly used treatment techniques included deep breathing exercises, relaxation techniques, incentive spirometry, thoracic mobility exercises, modified postural drainage, and range of motion exercises of the upper and the lower limbs. A smaller number of responders also practice percussion, vibrations, and coughing and huffing techniques.
3. The patients were made to dangle the lower limbs over the edge of the bed on postoperative day 1, along with common treatment techniques. Mobilization techniques like walking up to a chair, sit to stand exercises, and mobilization in the room were started on postoperative day 2. The patients were made to start with staircase climbing on the 4th postoperative day. The most regularly used functional evaluation test prior to discharge was the 6-minute walk test for thoracic surgery patients.

## 10. Preoperative and Postoperative Physiotherapy Assessment for Thoracic Surgery Patients

In our survey, the assessment of patients focused primarily on certain impairment measures such as physical examination, chest expansion, ABG analysis, pulmonary function test, chest X-ray, SpO_2_ (oxygen saturation), peripheral muscle strength, and cardiopulmonary exercise testing.

In the preoperative phase, physical examination should address the presence of dyspnoea, exercise tolerance, cough, and expectoration. Examination should also focus on respiratory rate, pattern of breathing, and wheezing. Patients may show either normal or altered breathing pattern on physical examination. Postoperatively, that is, after thoracic surgery, patients usually present with monotonous shallow breathing without spontaneous deep breaths, increase in respiratory rate, decreased tidal volume, and significant change in minute ventilation. Wheeze, rales, or prolonged breath sounds will be revealed on auscultation [8]. The current study shows that 95.2% of physiotherapists practiced physical examination as an assessment measure in their clinical settings.

Chest expansion is reduced in thoracic surgery patients, particularly on the side operated on. Clinically, the decreased chest movement may lead to hypoventilation and complications like atelectasis or lung collapse [9]. After pneumonectomy, the pulmonary artery and right ventricular pressures rise temporarily and then under normal circumstances return to the normal level. The ventilation of the remaining lung is increased by an increase in depth and rate of breathing. Due to changes in the thoracic volumes, the remaining lung gets adjusted to the change and becomes hyperinflated or expanded. In lobectomy, there is overinflation of contralateral as well as remaining ipsilateral lung tissue. This is because there is increased perfusion of the remaining lung and absolute reduction in the diffusion surface; that is, decrease in the ratio of dead space occurs with respect to total tidal volume which improves ventilatory efficiency [8]. The present study showed that 94.4% of the physiotherapists assessed chest expansion in their clinical settings.

Postoperatively, ABG analysis typically shows respiratory acidosis in the initial stages, while respiratory alkalosis develops in later stages [10]. During the immediate postoperative period, analgesics administered to relieve pain have a depressant effect on the central nervous system, thereby inhibiting ventilation. Hypoventilation, accompanied by the patients' reluctance or inability to cough, retention of secretions, and airway obstruction, causes atelectasis, leads to restriction in the exchange of gases across the alveolar capillary membrane, and causes CO_2_ retention. In a patient with sudden onset of alveolar hypoventilation causing a rise in PCO_2_, there would be a sudden fall in pH leading to uncompensated respiratory acidosis [11, 12]. 80.3% of the physiotherapists in the current survey considered ABG findings for assessment.

The chest radiographs of thoracic surgery patients show disease limited to one lobe or less [10]. Chest X-ray shows that the mediastinum either remains stationary or gradually shifts towards the pneumonectomy side which indicates atelectasis of the remaining lung or accumulation of fluid in the side operated on [13]. After lobectomy, there may be minor ipsilateral shift in the mediastinum or diaphragm, but a moderate to marked shift strongly suggests atelectasis in the remaining lobes, usually due to retained secretions or inability of the remaining lobes to reexpand fully [14]. On the PA view, there is triangular density behind the heart with loss of the medial portion of the left hemidiaphragm. On the lateral view, there is backward displacement of the oblique fissure and with increasing collapse there is increased density over the lower dorsal vertebrae [15]. Our survey showed that 93.5% of physiotherapists considered chest X-ray for assessment which is higher when compared to the extent to which this test is employed in a country like the United States of America (70%) [16].

Pulmonary function test is used to determine the degree to which the preexisting obstructive and restrictive components of pulmonary function may compromise the ability to ventilate adequately and to maintain clear lungs after thoracic surgery. Pulmonary function abnormalities in thoracic surgery include a decrease in forced expiratory volume, increased airway resistance, a decreased inspiratory capacity, and a decrease in maximum voluntary ventilation (MVV) [17]. The survey showed that 76.1% of the respondents used the pulmonary function test as assessment which is higher when compared to the extent to which this test is employed in countries like Australia and New Zealand (42.8%) [7].

Pulse oximetry (SpO_2_) is used to measure and record oxygen saturation. In thoracic surgery patients, there is a significant fall in PaO_2_, as well as abnormalities of gaseous exchange, disturbances in coordination of ventilation, and perfusion of lungs [17]. Pulse oximetry is used by 90.1% of the total responders which is comparable to the extent to which this technique is employed in countries like Australia and New Zealand which showed a response rate of 90.4% [7].

Peripheral muscle strength gradings were taken for flexion, abduction, extension, and internal rotation of the shoulder joint as these are some actions of the muscles which are divided during open thoracotomy. Postoperative physiotherapy exercise programme was provided to prevent or minimize shoulder dysfunction after thoracotomy [18]. In this study, 74.7% of the physiotherapists assessed peripheral muscle strength in their clinical settings.

## 11. Preoperative Physiotherapy Intervention for Thoracic Surgery Patients

Our survey found that preoperative physiotherapy techniques include deep breathing exercises, incentive spirometry, thoracic mobility exercises, huffing and coughing techniques, and ACBT techniques (more than 90%).

Deep breathing exercises are taught to the patients in the preoperative period. These can reduce the effects of an altered breathing pattern after thoracotomy and can help to obtain full expansion of the chest wall during spontaneous breathing. This is essential to help restore lung function and to prevent subsequent chest deformity. A large inflating volume and transpulmonary pressure gradient is needed to be maintained for several seconds in order to achieve lung reexpansion [19]. In this survey, 93.9% of physiotherapists practiced the administration of deep breathing exercises to preoperative patients which is a higher percentage than that of physiotherapists who administer the same exercises to patients in countries like Australia and New Zealand (87.5%) [7] and the United Kingdom (70%) [6].

Incentive spirometry is used to encourage sustained maximal inspiration, promote reinflation of lung tissue, and prevent or resolve atelectasis. Incentive spirometry involves deep breathing through a device with visual feedback, thought to maximize accuracy of breathing technique and motivation [19]. The results showed that 93.9% of the physiotherapists practiced incentive spirometry as physiotherapy interventions and this response rate is more than that indicated in a similar study carried out in countries like the United Kingdom (37%) [6] and Australia and New Zealand (42.8%) [7].

Coughing and huffing techniques are used in clearing secretions from large airways. The rapid, forceful expulsion of air from the lungs helps remove secretions from the large airways. In addition to mobilizing and expelling secretions, the high pressures generated during a cough may be an important factor in reexpanding lung tissue [20]. The current survey showed that 88.7% of physiotherapists practiced the coughing and huffing technique as a physiotherapy intervention. However, the response rate is lower than that reported in countries like Australia and New Zealand (94.2%) and the United Kingdom (89%) [6, 7].

Active cycle breathing exercises (ACBTs) are used in improving pulmonary function and airway clearance. The ACBTs are combinations of breathing control, thoracic expansion control, and forced expiratory techniques (FET). Breathing control has been referred to as diaphragmatic breathing, or as gentle breathing using the lower chest. Thoracic expansion exercises are simply active inspirations with larger than normal breaths, followed by relaxed expiration. This larger lung volume increases airflow through peripheral airways and collateral ventilation channels, which increases the gas volume available to mobilize secretions during expiration [20]. According to this survey, 86.7% of the physiotherapists practiced these patterns regularly. The response rate is more than that from countries like Australia and New Zealand (51.4%) and the United Kingdom [6, 7].

## 12. Postoperative Physiotherapy Management for Thoracic Surgery

The results of our survey showed that predominantly the same treatment techniques were employed postoperatively as employed preoperatively.

Breathing exercises are used to improve the efficiency of ventilation and gas exchange, increasing the excursion of the diaphragm and easing the mobilization of secretions [21]. Breathing exercises like localized breathing exercises, diaphragmatic breathing exercises, lateral basal expansion, upper lateral expansion, and apical pectoral expansion exercises are important for treating thoracic surgery patients. These exercises help to counteract an abnormal breathing pattern in the postoperative period [8]. According to our study, more than 95% of physiotherapists employ breathing exercises postoperatively and this is more than the response rate reported by physiotherapists in Australia and New Zealand (90%) [7].

Incentive spirometry has the same effect as shown in preoperative management. It is used primarily to prevent alveolar collapse and atelectasis in postoperative patients [19]. In the current study, it was noted that more than 90% of physiotherapists included spirometry as a physiotherapy intervention which is a higher percentage when compared to the percentage of physiotherapists who employ spirometry as intervention in countries like Australia and New Zealand (40%) [7]. The purpose of incentive spirometry is to increase the volume of air inspired.

Thoracic expansion exercises were given to improve the mobility of chest wall, trunk, and shoulder girdles. Exercises that combine stretching of these muscles with deep breathing improve ventilation on that side of the chest. These exercises are also used to reinforce or emphasize the depth of inspiration or controlled expiration [21]. According to this survey, the response rate of thoracic expansion exercises is 82.4% by physiotherapists in India. It is higher than the response rate reported in the United Kingdom (64.5%) [6].

Coughing techniques enhance the clearance of retained bronchial secretions, thus preventing atelectasis and infection. During a normal cough, airflow velocity varies in airways, creating high linear velocities, increased turbulence, and high shearing forces within the airway. These forces shear secretions from the airway walls, propelling them towards the larger airways and trachea which causes expelling of secretions [20]. Coughing techniques are practiced by 80% of the physiotherapists which is lower than the percentage of physiotherapists employing the same technique in countries like Australia and New Zealand (90%) [7].

Percussion is used to augment mobilization of secretions by mechanically dislodging viscous or adherent mucus from airways. Vibrations are used in conjunction with percussion to help move secretions to larger airways. The air cushion between the cupped hand and the chest wall generates the typical hollow sound, transmits the energy to the underlying lung, and helps mobilize secretions. The atelectatic areas of the lung can be reinflated and total lung/thorax compliance can be increased following percussion and vibration [8, 17]. In this survey, 66.6% of the physiotherapists practiced percussion and vibration in their clinical settings.

Positioning is advised for promoting relaxation, reducing postoperative pain, and causing lung expansion as also for draining secretions. In pneumonectomy, the lateral position is advised, as perfusion of the dependent lung is increased due to gravitational changes though ventilation is more difficult. The prone position is advantageous when a large quantity of bronchial secretions is present in the segmentectomy [8]. In this study, the results showed that 60% of physiotherapists practiced positioning technique as an intervention in their clinical settings.

Modified postural drainage (bronchial drainage) is used for airway clearance by mobilizing secretions from different lung segments to the central airways by placing the patient in various positions so that gravity assists in draining secretions. Postural drainage attempts to use gravity to move secretions from peripheral airways to the larger bronchi, from which they are more easily expectorated [8]. A total of 89.2% of the physiotherapists practice these patterns in their clinical settings as a physiotherapy intervention.

An upper limb exercise programme is commonly used to prevent or minimize shoulder dysfunction and to maintain joint range of motion after thoracotomy. Active shoulder exercises like flexion, abduction, external rotation and elevation, shoulder shrugging, and progressive shoulder and thoracic cage exercises are commonly used after thoracic surgery. Shoulder strengthening exercises for flexion, extension, abduction, and external rotations are usually performed after thoracic surgery [18]. Upper limb exercises are practiced by 74.8% of physiotherapists. This is a lower percentage than in Australia and New Zealand (93.5%) [7].

Lower limb exercises like hip and knee flexion and extension exercises and active leg, foot, and ankle exercises are taught to prevent circulatory problems like deep vein thrombosis and pulmonary embolism [21]. Thus, regular activation of the muscle pumps to minimize circulatory stasis and frequent body position changes is essential to reduce risks [22]. In the current survey, it was seen that 85.8% of physiotherapists practiced these patterns in their clinical settings.

Mobilization in the upright position coordinated with breathing control and supported coughing maneuvers is encouraged in order to reduce atelectasis and impaired mucociliary transport associated with surgery. Early mobilization elicits cardiopulmonary responses, resulting in enhancement of oxygen transport. These beneficial effects are enhanced by improved chest wall motion, improved gut mobility, and reduced intra-abdominal pressure. Also, a study stated that early walking has a good psychological effect on stable and beneficial breathing potentially leading to recovery of pulmonary function after surgery [23]. Extremity movement during ambulation increases alveolar ventilation, enhances ventilation and perfusion matching by increasing zone two of the lungs, and optimizes diffusing capacity through stimulating dilatation and recruitment of alveolar capillaries [22]. The current study showed that 74.3% of the responders practiced bedside sitting on the 2nd postoperative day. A similar study in Australia and New Zealand showed a response rate of 34.8% [7].

Room mobilization was started on the 2nd postoperative day by 71.1% of the responders and corridor mobilization was started on the same day by 39.8% of the responders. This response rate is lower than the response obtained in a similar study conducted in Australia and New Zealand (71.7%) [7]. The current survey showed that the most commonly used functional evaluation test is the 6-minute walk test employed by 77.4% of the physiotherapists. This test is used for evaluating the submaximal capacity, the effects of therapy, and the prognostic stratification of the patient [2].

## 13. Limitation of Study

One of the limitations of the study was that the majority of the responders were from Telangana, Karnataka, and Andhra Pradesh and this is not truly representative of India. Another possibility is that participants responded to the survey questionnaire with what they considered to be the best or most appropriate answer to each question. Therefore, their responses may not reflect the actual practice patterns of physiotherapists in India. And one more limitation is that we did not consider the minimally invasive thoracic surgery.

## 14. Conclusion

This survey provides an overview of current physiotherapy practices across India in thoracic surgeries. Commonly used assessment measures in the preoperative phase are physical examination, chest expansion, ABG analysis, pulmonary function test, chest X-ray, and SpO_2_ monitoring. Preoperative treatment techniques practiced are deep breathing exercises, huffing and coughing, active cycle of breathing exercises, incentive spirometry, and thoracic mobility exercises.

The most commonly used postoperative assessment measures are physical examination, chest expansion, ABG analysis, chest X-ray, pulmonary function test, and SpO_2_. The postoperative treatment techniques regularly used are breathing exercises, relaxation techniques, thoracic expansion exercises, incentive spirometry, coughing and huffing, and modified postural drainage. Mobilization which includes dangle, room and corridor mobilization, and the 6-minute walk test is commonly used as a functional evaluation test.

## Figures and Tables

**Table 1:** Professional profile of questionnaire responders.

Variables	Responses	Frequency (*n* = 234), *n*(%)
Qualification	BPT	100 (42.7)
	MPT	131 (56.0)
	PHD	3 (1.3)

Experience	6 months–1 year	62 (28.1)
	1-2 years	77 (34.8)
	2-3 years	28 (12.7)
	4–10 years	49 (22.2)
	>10 years	5 (2.3)

Type of surgical patient seen	Chest wall surgery	72 (30.8)
	Pulmonary resection	43 (18.4)
	Pleural surgery	31 (13.2)
	Others	79 (33.8)
	No response	9 (3.8)

Length of stay in hospital	0–3 days	22 (9.4)
	4–7 days	88 (37.6)
	8–10 days	97 (41.5)
	>10 days	15 (6.4)
	No response	12 (5.1)

**Table 2:** Preoperative assessment for thoracic surgery patients.

S. number	Techniques	Always or frequently, *n* (%)	Sometimes, *n* (%)	Seldom or never, *n* (%)
(1)	Measurement impairments			
	(a) Physical examination	218 (93.1)	12 (5.2)	1 (0.4)
	(b) Chest expansion	217 (92.7)	13 (5.6)	2 (0.9)
	(c) ABG analysis	183 (78.2)	37 (16.2)	7 (3.1)
	(d) Pulmonary function test	175 (74.1)	45 (19.6)	10 (4.3)
	(e) Chest X-ray	215 (93.5)	13 (5.7)	2 (0.9)
	(f) SpO_2_ (oxygen saturation)	211 (90.1)	16 (6.9)	4 (1.7)
	(g) Peripheral muscle strength	175 (74.7)	50 (21.6)	7 (3.0)
	(h) Cardiopulmonary exercise test	159 (67.9)	55 (23.8)	17 (7.3)
	(i) 6 min walk test, 12 min walk test	138 (60.0)	79 (34.3)	13 (5.7)

(2)	Measure of function			
	(j) Barthel index	157 (70.4)	38 (17.0)	28 (12.5)
	(k) Functional independence measure	164 (72.3)	41 (18.1)	22 (9.7)

**Table 3:** Preoperative physiotherapy interventions for thoracic surgery patients.

S. number	Treatment techniques	Always or frequently, *n* (%)	Sometimes, *n* (%)	Seldom or never, *n* (%)
(1)	Deep breathing exercises	217 (93.9)	13 (5.6)	1 (0.4)
(2)	Huffing and coughing	204 (88.7)	23 (10.0)	3 (1.3)
(3)	Active cycle of breathing exercises	203 (86.7)	26 (11.3)	2 (0.8)
(4)	Incentive spirometry	216 (92.3)	11 (4.8)	3 (1.3)
(5)	Thoracic mobility exercises	213 (91.0)	13 (5.7)	3 (1.3)

**Table 4:** Postoperative assessment for thoracic surgery patients.

S. number	Measurement	Always or frequently, *n* (%)	Sometimes, *n* (%)	Seldom or never, *n* (%)
(1)	Physical examination	223 (95.2)	7 (3.0)	3 (1.3)
(2)	Chest expansion	221 (94.4)	9 (3.9)	3 (1.3)
(3)	ABG analysis	198 (84.6)	30 (12.9)	4 (1.7)
(4)	Pulmonary function test	187 (79.9)	40 (17.2)	6 (2.6)
(5)	Chest X-ray	211 (90.1)	21 (9.1)	
(6)	SpO_2_ (oxygen saturation)	216 (92.3)	14 (6.0)	2 (0.9)

**Table 5:** Physiotherapy interventions used on the 1st postoperative day.

S. number	Techniques	Always or frequently, *n* (%)	Sometimes, *n* (%)	Seldom or never, *n* (%)
(1)	Chest physiotherapy			
	(a) Breathing exercises	221 (94.4)	7 (3.1)	2 (0.8)
	(b) Relaxation techniques	215 (91.8)	7 (3.1)	7 (3.1)
	(c) Thoracic expansion exercises	193 (82.4)	23 (10.1)	12 (5.3)
	(d) Incentive spirometry	198 (84.6)	18 (7.9)	13 (5.6)
	(e) Coughing & huffing techniques	177 (75.6)	34 (14.8)	19 (8.3)
	(f) Positioning	138 (58.9)	60 (26.3)	30 (13.2)
	(g) Modified postural drainage	206 (89.2)	21 (9.1)	4 (1.7)

(2)	Range of motion exercises			
	(a) Upper extremities	172 (74.8)	28 (12.2)	30 (13.2)
	(b) Lower extremities	201 (85.8)	23 (10.0)	7 (3.1)

(3)	Mobilization			
	(a) Dangle	198 (84.6)	21 (9.2)	10 (4.3)

**Table 6:** Physiotherapy interventions used on the 2nd postoperative day.

S. number	Techniques	Always or frequently, *n* (%)	Sometimes, *n* (%)	Seldom or never, *n* (%)
(1)	Chest physiotherapy			
	(a) Breathing exercises	228 (97.4)	1 (0.4)	2 (0.8)
	(b) Relaxation techniques	217 (92.7)	8 (3.5)	5 (2.2)
	(c) Thoracic expansion exercises	217 (92.7)	9 (3.9)	4 (1.8)
	(d) Incentive spirometry	217 (92.7)	12 (5.2)	1 (0.4)
	(e) Coughing & huffing techniques	192 (82.0)	28 (12.2)	10 (4.3)
	(f) Percussion & vibrations	156 (66.6)	53 (23.0)	21 (9.1)
	(g) Positioning	214 (91.4)	13 (5.7)	3 (1.3)
	(h) Modified postural drainage	179 (76.4)	33 (14.3)	18 (7.8)

(2)	Range of motion exercises			
	(a) Upper extremities	216 (92.3)	12 (5.2)	3 (1.3)
	(b) Lower extremities	211 (90.1)	14 (6.2)	3 (1.3)

(3)	Mobilization			
	(a) Dangle	174 (74.3)	30 (13.8)	14 (6.2)
	(b) Up to chair	156 (66.6)	56 (24.8)	14 (6.2)
	(c) Sit to stand	125 (53.4)	55 (25.1)	39 (16.9)

(4)	Ambulation			
	(a) Walking in the room	168 (71.7)	38 (16.6)	23 (13.1)
	(b) Walking in the corridor	86 (39.8)	69 (31.9)	61 (26.0)

**Table 7:** Physiotherapy interventions used on the 3rd postoperative day.

S. number	Techniques	Always or frequently, *n* (%)	Sometimes, *n* (%)	Seldom or never, *n* (%)
(1)	Chest physiotherapy			
	(a) Breathing exercises	228 (97.4)	3 (1.3)	1 (0.4)
	(b) Relaxation techniques	216 (92.3)	12 (5.2)	3 (1.3)
	(c) Incentive spirometry	226 (96.5)	5 (2.1)	2 (0.9)
	(d) Modified postural drainage	197 (84.1)	24 (10.3)	11 (4.7)

(2)	Range of motion exercises			
	(a) Upper extremities	222 (94.8)	8 (3.4)	3 (1.3)
	(b) Lower extremities	216 (92.3)	11 (4.7)	5 (2.1)

(3)	Mobilization			
	(a) Dangle	197 (84.1)	20 (9.0)	6 (2.7)
	(b) Up to chair	194 (82.9)	30 (13.0)	6 (2.7)
	(c) Sit to stand	177 (75.6)	38 (16.7)	12 (5.3)
	(d) Staircase climbing	86 (36.7)	61 (27.0)	79 (33.7)

**Table 8:** The mobilization on various postoperative days.

Postoperative day	Questions	Response	Frequency (*n* = 234), *n* (%)
Postoperative day 2	Distance walked by patient	25–50 feet	155 (66.2)
		60–70 feet	58 (24.8)
		80–90 feet	8 (3.5)
		No response	13 (5.6)

	Frequency of ambulation	1 time	3 (1.3)
		2 times	143 (61.1)
		3 times	17 (7.3)
		4 times	2 (0.9)

Postoperative day 3	Distance walked by patient	110–130 feet	158 (67.5)
		140–160 feet	58 (24.8)
		170–190 feet	12 (5.1)
		≥200 feet	4 (1.7)
		No response	2 (0.9)

	Staircase climbing	4th day	68 (29.1)
		5th day	113 (48.3)
		6th day	23 (9.8)
		7th day	13 (5.6)
		No response	17 (7.3)

	Functional evaluation test prior to discharge	2 min walk test	39 (16.7)
		6 min walk test	181 (77.4)
		12 min walk test	6 (2.6)
		Shuttle walk test	2 (0.9)
		No response	6 (2.6)
